# Spatiotemporal Acoustic Communication by a Single Sensor via Rotational Doppler Effect

**DOI:** 10.1002/advs.202206619

**Published:** 2023-02-03

**Authors:** Chuanxin Zhang, Xue Jiang, Jiajie He, Ying Li, Dean Ta

**Affiliations:** ^1^ Center for Biomedical Engineering School of Information Science and Technology Fudan University Shanghai 200433 China; ^2^ State Key Laboratory of ASIC and System Fudan University Shanghai 200433 China; ^3^ Underwater Communication Institute PengCheng Laboratory Shenzhen 518055 China; ^4^ Department of Rehabilitation Medicine Huashan Hospital Fudan University Shanghai 200040 China

**Keywords:** acoustic communication, rotational doppler effect, single sensor, spatiotemporal conversion

## Abstract

A longstanding pursuit in information communication is to increase transmission capacity and accuracy, with multiplexing technology playing as a promising solution. To overcome the challenges of limited spatial information density and systematic complexity in acoustic communication, here real‐time spatiotemporal communication is proposed and experimentally demonstrated by a single sensor based on the rotational Doppler effect. The information carried in multiplexed orbital‐angular‐momentum (OAM) channels is transformed into the physical quantities of the temporal harmonic waveform and simultaneously detected by a single sensor. This single‐sensor configuration is independent of the channel number and encoding scheme. The parallel transmission of complicated images is demonstrated by multiplexing eight OAM channels and achieving an extremely‐low bit error rate (BER) exceeding 0.02%, owing to the intrinsic discrete frequency shift of the rotational Doppler effect. The immunity to inner‐mode crosstalk and robustness to noise of the simple and low‐cost communication paradigm offers promising potential to promote relevant fields.

## Graphical abstract

A single‐sensor‐based simple and low‐cost communication paradigm to realize the real‐time and high‐capacity acoustic OAM multiplexing communication based on rotation Doppler effect. The exemption from spatial separation and intrinsic discrete frequency shift of the mechanism gives rise to the mitigation of crosstalk and robustness to noise, evidenced by the extremely‐low bit error rate exceeding 0.02%.

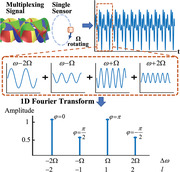

## 1 Introduction

Acoustic communication is of fundamental and irreplaceable significance in underwater exploration and other electromagnetic‐restricted applications where acoustic wave serves as the dominant information carrier due to the prohibitive long‐distance propagation of the electromagnetic wave.^[^ ^1^ ^]^ However, the relatively lower frequency and propagation speed of acoustic waves hinder the improvement of the communication speed and data capacity,^[^ ^2^ ^]^ which is essential to satisfy the substantially increasing requirement of high‐definition signal transmission in practice. Multiplexing technology has been proposed as an efficient solution to promote the transmission capacity in acoustic communication,^[^ ^3^ ^]^ including frequency‐division multiplexing, time‐division multiplexing, and so on. As an emergent branch of multiplexing technology, orbital‐angular‐momentum‐division multiplexing^[^ ^4^, ^5^, ^6^, ^7^, ^8^ ^]^ based on acoustic vortex beams^[^ ^9^, ^10^, ^11^, ^12^, ^13^ ^]^ has attracted broad attention owing to its intrinsic orthogonality, infinite dimensionality of its Hilbert space and compatibility with existing multiplexing technologies. As a consequence, each vortex beam of different topological charge *l* plays as an orthogonal OAM channel,^[^ ^6^ ^]^ which can boost the transmission capacity by integrating with other state‐of‐the‐art communication technologies, for example, the combination of OAM and multiple input multiple output technologies^[^ ^14^ ^]^ has been proposed to further increase the communication speed, and mitigate the crosstalk with the equalization algorithm.^[^ ^15^, ^16^, ^17^ ^]^

So far, existing acoustic OAM multiplexing communication links can be classified into the active field‐scanning‐based scheme or the passive metamaterial‐based scheme. In the active scheme, the information carried in a specific OAM channel is extracted from the 2D inner product between the multiplexed field and individual OAM base. As the 2D multiplexed field should be measured with the time‐consuming point‐by‐point scanning or sensor array of complex hardware and inadequate spatial resolution, the active scheme is challenged to realize the real‐time signal transmission in a simple configuration. By spatially separating the OAM modes and detecting the information carried in each OAM channel with a specific sensor, the passive scheme can support real‐time communication while suffering from insertion loss, diffraction effect, signal overlapping, and increasing complexity. It poses a fundamental limitation on the further enhancement of spatial information density since the sensor's number should be consistent with the number of OAM channels. Therefore, it remains an open problem to reconcile the contradiction between the system complexity and the transmission capacity for the currently available acoustic OAM communication technologies. One can expect that the real‐time and high‐speed acoustic communication with a single sensor independent of the number of OAM channels, would profoundly increase the spatial information density and relieve the hardware burden.

The concept of single‐sensor measurement is widely recognized as a result of compressive sensing^[^ ^18^, ^19^ ^]^ and finds intriguing applications in imaging^[^ ^20^, ^21^ ^]^ and source localization.^[^ ^22^, ^23^ ^]^ Although compressive‐sensing‐based single‐sensor detection can significantly reduce the hardware complexity and processing cost, it suffers from a time‐consuming problem since multiple measurements and convex optimization are required. As a consequence, the compressive‐sensing‐based single‐sensor paradigm is nonoptimal to live up to the expectation of real‐time communication. On the contrary, we consider to find a solution from the basic characteristics of the OAM itself. One of the phenomena stemming from OAM is the rotational Doppler effect.^[^ ^24^, ^25^ ^]^ It tells that an *l*th vortex beam interacting with a rotating object of angular frequency Ω will acquire the Doppler frequency shift of Δ*ω* = *l*Ω.^[^ ^26^, ^27^ ^]^ The basic mechanism of rotational Doppler effect has been demonstrated with acoustic waveguides at 100 Hz to access the extreme Doppler shift,^[^ ^28^ ^]^ and it has also been used in mimicking the Penrose superradiance,^[^ ^29^, ^30^ ^]^ cooling and heating,^[^ ^31^ ^]^ and measuring rotating objects.^[^ ^27^ ^]^ Importantly, the rotational Doppler effect builds the bridge between the angular momentum and frequency. A mode‐frequency‐division method has been proposed by rotating the OAM sources to generate multiple frequency signals,^[^ ^4^, ^32^ ^]^ where the harmonic frequency waves may asynchronously propagate due to the dispersion.^[^ ^33^ ^]^ Apart from the rotating source, we recognize that the vortex beams of identical frequency but different OAM orders can play as the multiplexed carries, which can be efficiently separated in the frequency domain and simultaneously measured by a single sensor, getting rid of the field‐scanning, insertion loss, spatial overlapping, and propagation dispersion.

In this work, we theoretically propose and experimentally demonstrate the real‐time spatiotemporal acoustic communication with a single sensor based on the rotational Doppler effect. The spatiotemporal conversion between the OAM mode in the spatial domain and the harmonic frequency embedded in a temporal waveform gives rise to single‐sensor‐based communication independent of the number of multiplexed OAM channels. This single‐sensor‐based paradigm relieves the hardware and time burdens in the active inner‐product‐based method, and it also breaks the limitation on the maximum available OAM channels in the passive metamaterial‐based method. On this basis, we realize the multiplexing communication with eight OAM channels of the order from −4 to 4. The combination with both amplitude‐shift keying (ASK) and phase‐shift keying (PSK) modulation technologies is validated, and real‐time parallel transmission of eight complicated images is demonstrated. We also analyze the transmission speed and accuracy of the single‐sensor‐based spatiotemporal communication mechanism. Benefited from the intrinsic discrete frequency shift of the rotational Doppler effect together with the break of the dependence between BER and sensors number, an extremely‐low BER exceeding 0.02% is realized in our experiments, proving the immunity to inner‐mode crosstalk and robustness to ambient noise. Our mechanism exempting from the field‐scanning and limitation on maximum OAM channels endows real‐time, high‐capacity, and high‐accuracy communication in a simple and low‐cost passive configuration, providing the potential to enhance the transmission speed and far‐reaching impact on relevant fields.

## 2 Result

### 2.1 Models and Theory

**Figure 1:**
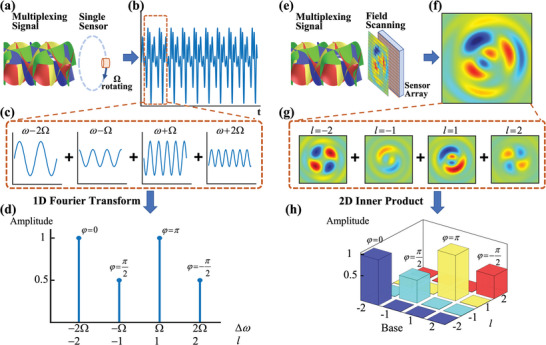
Comparison of different acoustic OAM communication mechanisms. a) Schematic illustration of the real‐time OAM‐based acoustic spatiotemporal communication with a single rotating sensor. b) Temporal signal received by the sensor, which contains different frequency components c) derived from the distinct OAM channels via the rotational Doppler effect. d) The amplitude and phase spatially encoded in different OAM channels are directly extracted from the temporal signal through the 1D fast Fourier transfer. e) Schematic illustration of the traditional OAM communication based on filed scanning method by sensors array. f) The multiplexed acoustic pressure field (real part) measured by field scanning, where vortex beams serving as different OAM channels are superimposed (g). h)The information encoded in different OAM channels should be decoded by performing the 2D inner product between the multiplexed acoustic field and the orthogonal OAM bases.

The schematic diagram of the proposed acoustic spatiotemporal communication by a single sensor via the rotational Doppler effect is illustrated in **Figure** **1**a–d, side by side with the conventional OAM communication method based on field scanning and inner product operation (Figure 1e–h). Acoustic vortex beams of different topological charge *l* are used as the orthogonal OAM channels to encode information, which is subject to parallelly propagate in space. The multiplexed acoustic pressure field superimposed with different spatial modes on the transverse plane is expressed as

(1) $$p\left(r,\theta ,t\right)=\sum_{l}A_{l}\left(t\right)e^{i\left(\omega t+l\theta +\varphi_{l}\left(t\right)\right)}$$

where *A_l_* (*t*) and *φ_l_* (*t*) represent the time‐dependent data stream encoded on the amplitude and phase of the *l*th vortex beam, through the ASK and PSK modulation technologies. Considering the orthogonality of the OAM spatial modes, a direct demultiplexing method in the receiving terminal is performing the inner product operation between the superimposed field and the orthogonal OAM base,^[^ ^34^ ^]^ by which the specific OAM component can be separated to decode the amplitude and phase information, as demonstrated in the previous acoustic OAM communication scheme.^[^ ^6^ ^]^ Therefore, it is necessary to measure the 2D field distribution with the time‐consuming field scanning, where the acquisition and analysis of 2D spatial signal tremendously increase the complexity in hardware architecture, signal processing, and energy consumption.

In our proposed spatiotemporal acoustic communication system, a single rotating sensor is employed to receive the multiplexed 1D temporal signal and directly demultiplex the spatially superimposed OAM channels based on rotational Doppler effect, exempted from the field‐scanning, complex signal processing, insertion loss in the cascaded multilayer metasurface and overlapping in the diffraction‐based separation.^[^ ^5^, ^7^, ^35^, ^36^ ^]^ The temporal signal received by the sensor rotated around the OAM axis at the angular frequency *Ω* can be expressed as^[^ ^28^ ^]^

(2) $$p\left(t\right)=\sum_{l}A_{l}\left(t\right)\sin\left[\left(\omega +l\Omega \right)t+\varphi_{l}\left(t\right)\right]$$

which contains harmonic frequency components (Figure 1b,c). It is observed in Equation (2) that an angular frequency shift *l*Ω is introduced to the spatially multiplexed *l*th vortex beam, building up the bridge between the OAM and frequency domains and rendering the spatiotemporal communication by a single sensor. On this basis, the information encoded in the amplitude *A_l_* (*t*) and phase *φ*_*l*_(*t*) of the specific vortex beam can be expediently decoded by simply performing the 1D fast Fourier transfer (FFT) of the temporal signal and reading the amplitude/phase on the particular frequency component (Figure 1d).

### 2.2 Generation of Perfect Vortex Beam

The first step of real‐time spatiotemporal communication is generating the multiplexed vortex beams of different orders and encoding the targeted information by dynamically modulating the amplitude and phase of the vortex beams. The vortex beams of different OAM orders are characterized by distinct intrinsic intensity profiles along the radial direction, typically, the higher the absolute OAM orders, the larger the diameter of the first intensity peak.^[^ ^37^ ^]^ These discrepant intensity profiles of the multiplexed spatial modes lead to the *l*‐dependent diffraction effect during the signal propagation and inconsonant maximum power position, which hinder the convenient detection of the receiving terminal and further enhancement of transmission capacity by increasing the OAM channels.^[^ ^38^ ^]^ We introduce the perfect vortex beams as the multiplexed information carriers which share the identical intensity profile independent on *l*,^[^ ^39^, ^40^ ^]^ and therefore diffract in a similar manner during the transmission in free space. The employment of iterative optimization (details in Supporting Information) ensuring perfect vortex beam as the information carrier contributes to efficient communication with a single sensor. We generate eight perfect vortex beams (from −4th to 4th) as the multiplexing OAM channels with an airborne phased array of 16 × 16 elements (MSO‐P1040H07, 40 kHz).

### 2.3 Experimental Realization of Real‐Time Spatiotemporal Communication

**Figure 2:**
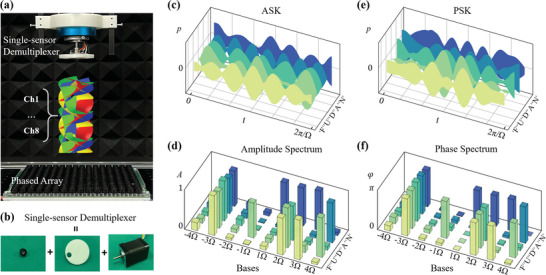
Experimental results of the OAM‐based acoustic spatiotemporal communication by a single sensor. a) Photograph of the experimental setup for the real‐time acoustic spatiotemporal communication system. The data streams are continuously transmitted through the multiplexed vortex beams which are dynamically manipulated with the phased array (40 kHz). A single rotating sensor of angular frequency Ω is placed in the receiving terminal to receive and demultiplex the signals via the rotational Doppler effect. b) The single‐sensor‐based demultiplexer consists of a compact ultrasound sensor, a supporting baffle, and an electric motor that enables stable rotation. Binary (ASCII) representations of the letters in the word FUDAN are transmitted through different OAM channels (*l* from −4 to +4), with the information encoded in the amplitude c,d) and phase e,f) of the multiplexed vortex beams. Received temporal signals corresponding to each letter in FUDAN by the single sensor, with c) the ASK and e) the PSK modulation technologies. d,f) Binary representations of the letters reconstructed from the temporal signals.

We integrate the spatiotemporal communication with both the ASK and PSK modulation technologies to demonstrate the real‐time and high‐capacity performance, where eight parallel data streams are translated into the amplitude (phase) values of 1 (*π*) or 0 on eight OAM channels according to the binary coding principle. As an essential process, demultiplexing which retrieves the information from the superimposed signal determines the ultimate transmission speed and accuracy of the system.^[^ ^35^ ^]^ We construct a single‐sensor demultiplexer that enables the simultaneous demultiplexing and the recording of parallel data streams with a single sensor. Photographs of the experimental setup and the single‐sensor demultiplexer are shown in **Figure** **2**a,b. The sensor mounted on a supporting baffle is rotated by an electric motor driven at a constant speed. In the first experimental demonstration, we encode the word FUDAN in ASCII binary protocol into eight OAM channels with the ASK and PSK modulation technologies, where each OAM mode carries a 1‐bit component of the 1‐byte binary representation of the letters in every signal cycle time (2*π*/Ω). Therefore, a letter (1 byte) is transmitted in a signal cycle through eight OAM channels. The sensor is rotated at the angular frequency 20*π* rad s^−1^ around the OAM axis and placed 200 mm away from the phased array. The measured temporal signal at the receiving end by the single sensor for different letters is plotted in Figure 2c,f, under the ASK and PSK modulations. The temporal signal contains multiple frequency components derived from the multiplexed OAM spatial modes, and the information carried in different channels is retrieved with the 1D FFT of the temporal signal. This polychromatic waveform presents different envelopes, or so‐called beats, which are analogous to the beat frequency in the fluid velocity measurement based on the linear Doppler effect.^[^ ^41^ ^]^ The amplitude and phase spectrum of the experimentally measured temporal signals are plotted in Figure 2d,f, where the binary representations of the letters are explicitly displayed and agree well with the target information. The slight spectrum leakage to other bases is mainly ascribed to the inadequate precision and spatial resolution of the phased array in generating the multiplexed vortex beams, as we discuss in Supporting Information S1. As a comparison, we also perform the results with the active method by calculating the 2D inner product between the multiplexed field and the orthogonal spatial bases, where similar spectrum leakage appears.

**Figure 3:**
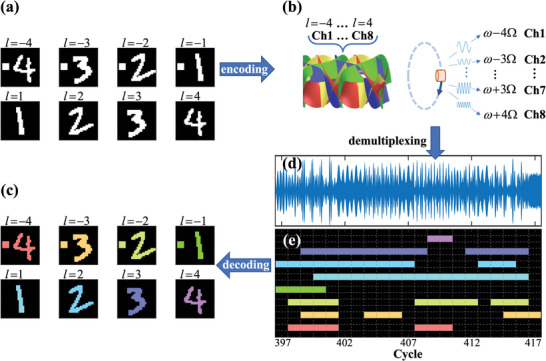
Real‐time acoustic spatiotemporal communication in eight multiplexed OAM channels by a single sensor. a) Eight images of the numeric values from –4 to 4 are encoded parallelly to the OAM channels (*l* from −4 to 4). b) The data streams are continuously transmitted by dynamically manipulating the multiplexed vortex beams and detecting the temporal signal by a single sensor. c) Temporal signal (partially plotted within 397 to 417 cycle) received by the sensor, which contains eight frequency components demultiplexed from eight OAM channels. d) Short‐time Fourier spectrum of the temporal signal in (c), where the data streams in different OAM channels (i. e., different harmonic frequency shifts) are plotted in a different color. e) Reconstructed images from the received data streams in eight OAM channels.

The fast, simple, and accurate merits of the proposed single‐sensor‐based communication give rise to the real‐time and parallel transmission of high‐speed data streams in an extremely simple configuration. We demonstrate the performance by parallelly and continuously transmitting eight binary numeral images (each consisting of 28 × 28 pixels, **Figure** **3**a) through eight OAM channels (*l* from −4 to 4). We use the ASK modulation as a demonstration. The angular frequency Ω of the sensor is increased to 60*π* rad s^−1^, and the signal cycle time is set to be 0.05 s. The received temporal signal within the 397 to 417 cycle is plotted in Figure 3c, and the entire temporal signal is provided in Supporting Information S1. The data stream carried in the *l*th OAM channel is decoded by performing the short‐time FFT and extracting the discrete frequency components at 40 kHz ± *l* × 30 Hz, as plotted in Figure 3d, from which eight numeral images are accurately reconstructed (Figure 3e). Our approach shows that real‐time acoustic OAM communication can be conveniently realized with a single sensor, without the need for a sensor array or metamaterial. As a general method, the same communication equipment can work for any frequency band and OAM order, without the need of redesigning the system when changing the communication parameters. In addition, our approach is also compatible with other OAM launch systems (e.g., phased array and metamaterial) and can be expanded into wherever OAM extraction is needed.

### 2.4 Analysis of Transmission Efficiency and Accuracy

The transmission speed in our spatiotemporal multiplexing communication method is eight times that of a single channel. In our experiments, the data capacity of 160‐bit s ^−1^ is achieved. The data capacity in our communication link is limited by the available phased array and the electric motor in the laboratory. A larger number of parallel spatial modes of higher OAM order can be generated with the phased array of finer modulation precision, and the signal cycle time can be dramatically reduced by employing the faster electric motor or applying an advanced harmonic analysis algorithm.^[^ ^42^ ^]^ Meanwhile, as a completely independent degree of freedom, OAM‐based communication can be integrated with other state‐of‐the‐art communication modulation technologies to further enhance the data capacity. We can estimate that by applying for the OAM orders from −16 to 16, integrating with the quadrature phase‐shift keying modulation, and reducing the signal cycle time to 0.005 s with the commercially available electric motor, an aggregate data capacity of 25.6‐kbit s ^−1^ can be obtained. In addition, it is noteworthy that only a single sensor is needed in spite of the number of multiplexed modes, in contrast to the previous field‐scanning‐free OAM communication methods where each spatial channel requires a sensor for specific detection.^[^ ^5^, ^7^, ^35^, ^36^ ^]^ Therefore, this single‐senor‐based communication mechanism shows prominent advantages to boost the transmission capacity by multiplexing a larger number of OAM channels, without increasing the burdens in the hardware system and signal‐processing procedure.

**Figure 4:**
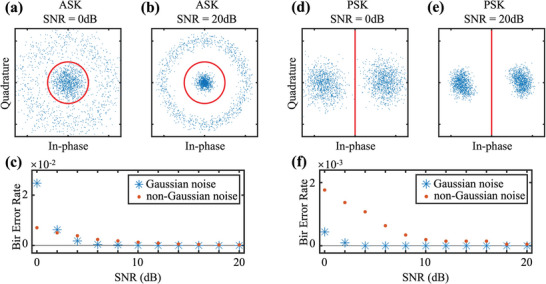
Error analyses of the OAM‐based acoustic spatiotemporal communication by a single sensor. Constellation diagrams of the measured signal under a,b) ASK and d,e) PSK modulations, with the SNR being 0 and 20 dB. c,f) Relationship between the BER and SNR with Gaussian and non‐Gaussian noises, under ASK and PSK modulations, respectively.

We finally give the quantitative estimation of the transmission accuracy of the single‐sensor‐based spatiotemporal communication system. In practical scenarios ambient noise is a critical factor to affect transmission accuracy. The discrete frequency shift induced by the rotational Doppler effect innately forbids the spectrum broadening which is intractable while a common problem in low signal‐to‐noise ratio (SNR) environments. It also prominently eliminates the crosstalk among OAM channels since it is unnecessary to spatially separate the vortex beams, and therefore, avoids the energy overlapping among different modes. We introduce the BER to characterize the communication performance in different SNR conditions, in each of which we repeat 10 times 256 combinations (total number of possible combinations for eight channels) to evaluate the BER. The SNR is controlled by increasing the noise amplitude at the emission end, and the constellation diagrams of the received signal in the 0 and 20 dB SNR situations are drawn in **Figure** **4**a,b, under the ASK and PSK modulation for the Gaussian noise. The constellation diagram represents the signal as a polar vector in the complex plane, in which the distance *r* from the original point denotes the amplitude of the signal, and the angle *θ* denotes the phase of the signal. Therefore, signal with different amplitude or phase information appears at different positions, from which the crosstalk and bit error can also be clearly recognized. As a demonstration, 256 combinations (256 × 8 scatters) are drawn in the constellation diagram. The threshold to determine the binary value from the signal amplitude is set to *r* = 0.5 (red line in Figure 4), i. e., the signal located outside the ring is set to binary value 1. The binary values of 0 and 1 are clearly distinguished in the 20 dB SNR condition. The ambient noise has a more serious impact in the 0 dB SNR condition and the crosstalk is more obvious (Figure 4a). A similar result is observed in the PSK modulation as shown in Figure 4b, where the threshold to distinguish the binary phase is set to be *φ* = 0. To show a clear evolution of the BER with varying ambient noise, we calculate the summation of BERs in eight OAM channels as a function of SNR for both the Gaussian and non‐Gaussian noises under the ASK and PSK modulations, as plotted in Figure 4c,d. As a typical example of non‐Gaussian noise, we consider impulsive noise which is described by *α*‐stable noise model^[^ ^43^ ^]^ and widely used in describing many noisy conditions. It is observed when the SNR is larger than 2 dB, the transmission accuracy is higher than 99% (BER < 1%) for ASK modulation, and it reaches 99.98% (BER < 0.02%) for PSK modulation. The relatively lower BER in PSK modulation has been recognized in the traditional communication methods^[^ ^44^ ^]^ and it is also verified in our spatiotemporal communication system, which can be intuitively explained by the larger distance between the two target signals in the constellation diagram and therefore stronger robustness to noise interference. In addition to ambient noise, the alignment between the propagation direction and rotation axis of the sensor is important to affect communication accuracy. Discussion on the influence of alignment and an adaptive calibration method are provided in Supporting Information S1.

## 3 Conclusion

As a completely independent degree of freedom, OAM opens an avenue to a high‐capacity communication paradigm, but acoustic OAM communication is still in an early stage. Here, we propose and experimentally demonstrate the real‐time spatiotemporal acoustic communication by a single sensor based on the rotational Doppler effect, exempting from bulky and complex devices, insertion loss, spatial overlapping, and propagation dispersion. The integration with both the ASK and PSK modulation technologies is validated, and real‐time parallel transmission of eight complicated images is demonstrated. Benefiting from the intrinsic discrete frequency shift of the rotational Doppler effect, an extremely‐low bit error rate exceeding 0.02% is realized, proving the superior immunity to inner‐mode crosstalk and robustness to ambient noise. It is noteworthy that different from the single sensor detection based on compressive sensing, our method arises from the innate physical property of the OAM beam and is analytically guided. In addition, only one measurement is needed without any time‐consuming operation such as solving the convex optimization. Our approach lends new perspectives on OAM communication and offers the possibility of finding more extensive applications related to OAM.

To further increase the transmission capacity by multiplexing more OAM channels, the major challenge is dynamically generating more vortex beams of higher order, where a phased array consisting of massive independent elements is required. Both high spatial resolution and synchronization precision are critical factors. In our experiments, a phased array with 16 × 16 elements can generate eight multiplexing vortex beams with the help of the developed iterative optimization method, and more vortex beams can be synthesized by introducing a phased array with larger aperture or increasing its spatial resolution. It is noteworthy that at the receiving end, there is in principle no limitation on the mode number since all OAM modes are endowed with distinct frequency shifts and simultaneously detected by the single sensor. In such a case, the frequency response of the sensor should be wide enough to guarantee the detection of all frequency components.

## Conflict of Interest

The authors declare no conflict of interest.

## Supporting information

Supporting Information

## Data Availability

The data that support the findings of this study are available from the corresponding author upon reasonable request.
