# Supplementary material for: Spatiotemporal Acoustic Communication by a Single Sensor via Rotational Doppler Effect
Source: Adv Sci (Weinh). 2023 Feb 3;10(10):2206619. doi: 10.1002/advs.202206619 (PMC10074052; doi:10.1002/advs.202206619)
Supplement: Supplementary file 1 — Supporting Information [file ADVS-10-2206619-s001.pdf]

Supplementary Material

Real-time Spatiotemporal Acoustic Communication by a Single Sensor via Rotational Doppler Effect

Chuanxin Zhang[1,2,*], Xue Jiang[1,2,4,*,‡], Jiajie He[1,2], Ying Li[1], and Dean Ta[1,2,3,‡]

[1]Center for Biomedical Engineering, School of Information Science and Technology, Fudan University, Shanghai, 200433, China

[2]State Key Laboratory of ASIC and System, Fudan University, Shanghai, 200433, China

[3]Department of Rehabilitation Medicine, Huashan Hospital, Fudan University, Shanghai, 200040, China

[4]PengCheng Laboratory, Shenzhen, 518055, China

[*]These authors contributed equally to this work

[‡]Email: xuejiang@fudan.edu.cn; tda@fudan.edu.cn

CGH-based algorithm for ultrasound perfect vortex beams

For facilitating the proof-of-concept experiment, we use an airborne phased array (40 kHz) which consists of 16×16 transducers to accomplish the OAM multiplexing procedure with perfect vortex beams. Compared with traditional vortex, the perfect vortex beam has the radial intensity profile independent of orbital angular momentum (OAM). The phased array generates the multiplexed perfect vortex beams of different OAM order l (topological charge), whose amplitude and phase are dynamically manipulated.

There are two issues required to be considered for the superposition and modulation of ultrasound perfect vortex beams in this work. On the one hand, the spatial resolution on the array is insufficient (larger than one wavelength) to generate the high-accuracy ultrasound field due to the finite transducer size (about 10 mm for the available commercial transducer). On the other hand, the perfect field construction generally requires modulating both ultrasound amplitude and phase on the emitting surface, while the modulation of hundreds of transducers on the array tremendously burdens the hardware. To solve the dilemma, we developed an improved iteration algorithm to precisely construct the desired ultrasound filed by modulating only the ultrasound phase on the array, with the transducers' size larger than one wavelength.

Here, we introduced computer-generated holography (CGH) based method to generate the perfect vortex beams. Gerchberg–Saxton (GS) algorithm has been widely used to calculate the holographic phase profile on the source plane for constructing the 3D field. The phase profile can be retrieved by propagating and backpropagating between the target and the source plane iteratively. Different from the random intensity pattern, acoustic vortex carrying OAM is manifested as helical phase fronts with an azimuthal phase term $e^{il\varphi}$, where φ is the azimuthal angle. Sound pressure field can be decomposed into multiple angular harmonics and analyzed with the angular spectrum:

$$\frac{1}{2}\int_0^{2\pi} e^{il\varphi} e^{il'\varphi} \mathrm{d}\varphi = \begin{cases} 1, l = l' \\ 0, l \neq l' \end{cases}$$

$$p(r,\varphi,z)=\frac{1}{N}\sum_{l=0}^{N-1}A(r,l,z)e^{i\frac{2\pi}{N}l\varphi}$$

At the begin of the iteration, the initial target boundary conditions are defined from the angular spectrum, where $A(r, l, z)$ is settled by the desired modulation. Numerically backpropagated the desired beams and get the boundary condition on the source plane. The source boundary condition is modified by binarizing the ultrasound amplitude into one or zero, and quantifying the ultrasound phase with the resolution of $\pi/16$ radians to keep consistence with the HPA hardware. The modified source condition generates the vortex beams by propagating the wave. Traditionally, the target boundary condition of the desired field is corrected by directly replacing the amplitude of the desired ultrasound field. In our improved iteration algorithm, the target boundary condition is adjusted in the angular frequency domain. We modify the spectral amplitude and phase of each OAM order at the desired radius which allows the construction of perfect vortex beams and improves the accuracy with the “insufficient” and phase-only-modulation array used in the experiments. We repeated these steps to iteratively optimize the phase modulation on the source plane. Typically, 30 times of iteration are required for constructing the ultrasound fields demonstrated in this work. The iteration optimization processes are as follows:

(1) Propagate the field from the source plane to generate the vortex beams;

(2) Transfer the beams into angular frequency domain. Adjust the amplitude/phase of each OAM order;

(3) Back-propagate the beams to the source plane;

(4) Binarized the amplitude and weight the discrepancy on the source plane; Quantify the forward-propagated phase profile;

(5) Break or repeat from step (1).

Comparison with the inner-product method

We encode the word FUDAN in ASCII binary protocol into the eight OAM channels with the ASK and PSK modulation technologies. Under the ASK/PSK modulation, we constraint the amplitude/phase of each OAM order l during the iteration. In order to visualize the perfect vortex beams, we simulate the acoustic field distributions. The simulation resembles the experimental setup. The simulated acoustic field distributions under ASK modulation and PSK modulations are plotted in Figs. S1 and S2 (pressure amplitude in (a) and pressure phase in (b)). The information encoded in specific OAM channel is extracted from the two-dimensional (2D) inner-product between the multiplexed field and individual OAM base, as plotted in Fig. S1(c) (ASK) and Fig. S2(c) (PSK). Similar spectrum leakage is observed, proving that the leakage is not resulted from the single-sensor-based communication method proposed in our work, but from the inadequate precision and spatial resolution of the phase array in generating the multiplexed vortex beams. To verify the validity of perfect vortex beams, we further calculate the intensity distribution along radial direction, as plotted in Fig. S1(d) and S2(d). Intensity peaks occurs at r = 20 mm despite of the different modulation and carried information, which gives inspiration for the future acoustic OAM communication technology.

Influence of alignment

A good alignment is critical to guarantee the transmission accuracy in the OAM multiplexing communication. The quantitative evaluation of the alignment influence obtained from the experimental results are plotted in Fig. S3. The misalignment degree is defined by the deviation angle α with respect to the propagating direction (Fig. S3 (a)). The experimentally measured BERs under the ASK and PSK modulations in 8 multiplexing channels as a function of α are plotted in Figs. S3 (b) and (c), respectively.

It is observed that the PSK modulation is more robust to the deviation which is similar to the performance in noise environment. When the misalignment angle is smaller than 0.3° (1°), the BER of ASK (PSK) remains zero to maintain a relatively accurate communication performance. The increase of misalignment would result in nonnegligible communication error. In such a case, we have also developed an adaption correction method to adjust the deviation. Instead of moving the rotation center by the mechanical motor, the phased array adaptively adjusts the center of the multiplexing vortex beams by modulating the emitted phase to find the maximum signal, which enables the rapid and effecient alignment while keeping the whole configuration stationary. The misalignment angle in our system is much lower than 0.2° which guarantees the sufficient transmission accuracy for both ASK and PSK modulated signal.

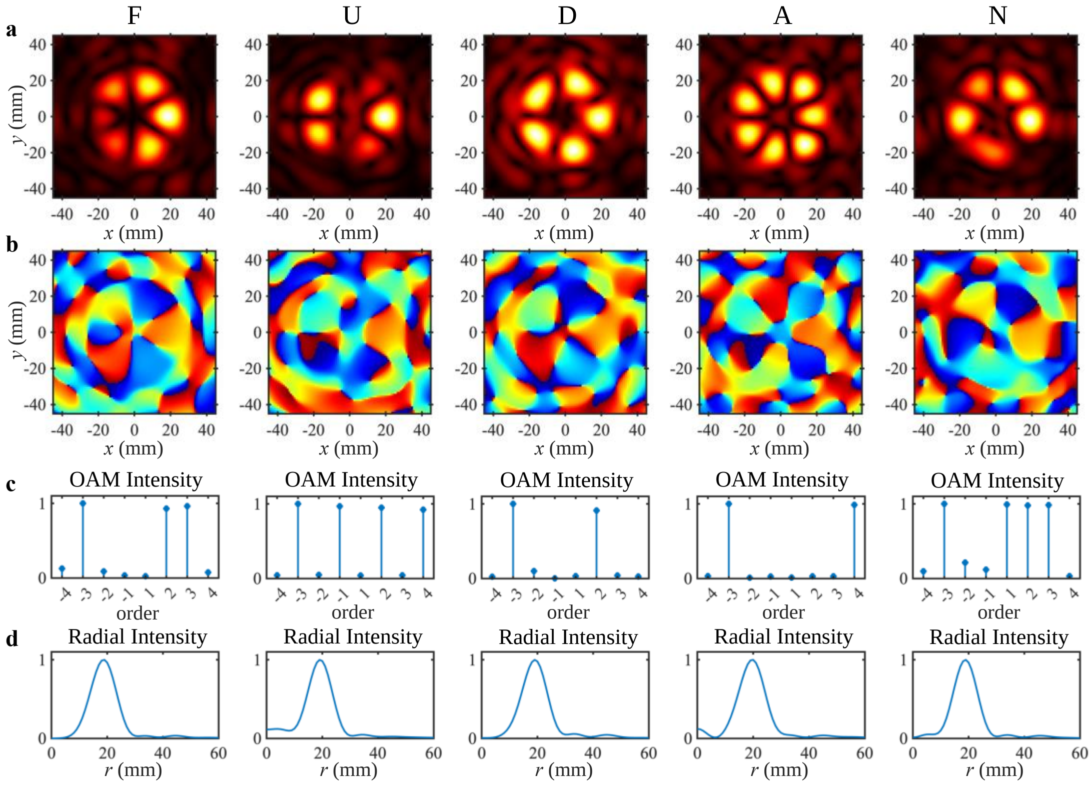

Figure S1. Amplitude modulation of acoustic perfect vortex based on CGH. The multiplexed acoustic pressure amplitude (a) and phase (b) fields corresponding to each letter in FUDAN. (c) Intensity in different OAM channels (*l* from -4 to +4), calculated with inner product between the entire 2D field and individual OAM bases. (d) Radial intensity profile. The intensity peaks at $r = 20$ mm despite of the different modulation method and different carried information.

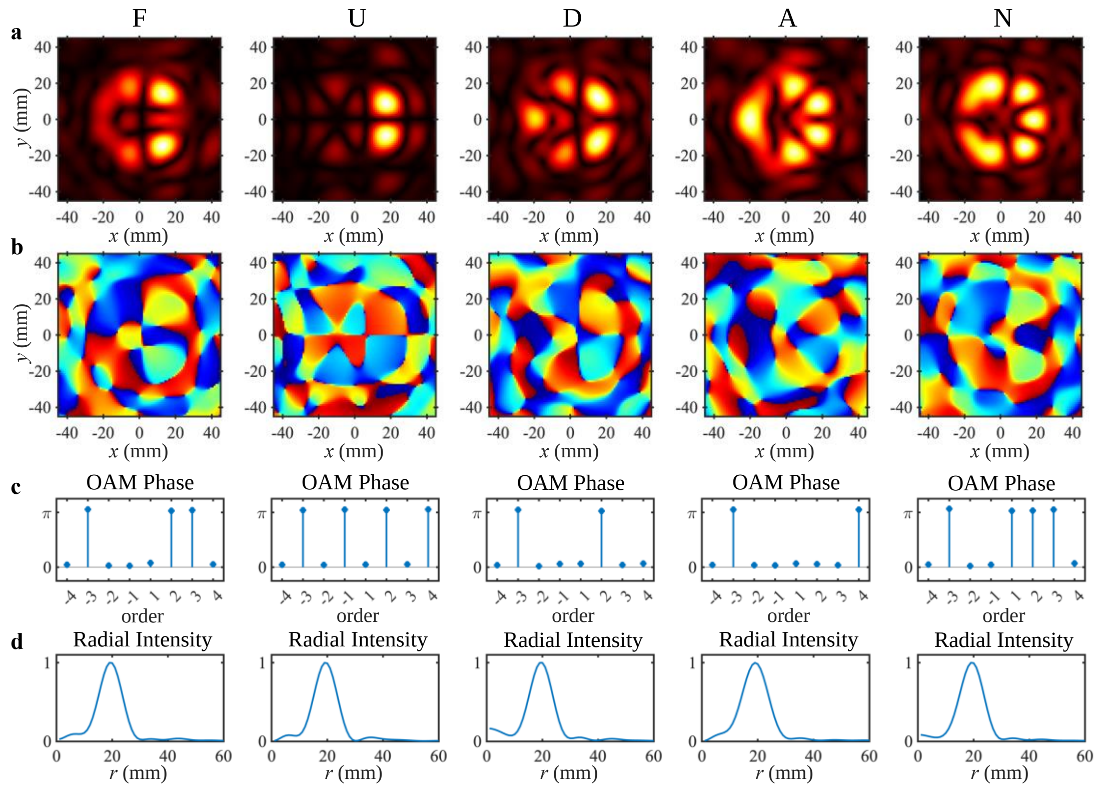

Figure S2. Phase modulation of acoustic perfect vortex based on CGH. The multiplexed acoustic pressure amplitude (a) and phase (b) fields corresponding to each letter in FUDAN. (c) Phase in different OAM channels (*l* from -4 to +4), calculated with inner product. (d) Radial intensity profile.

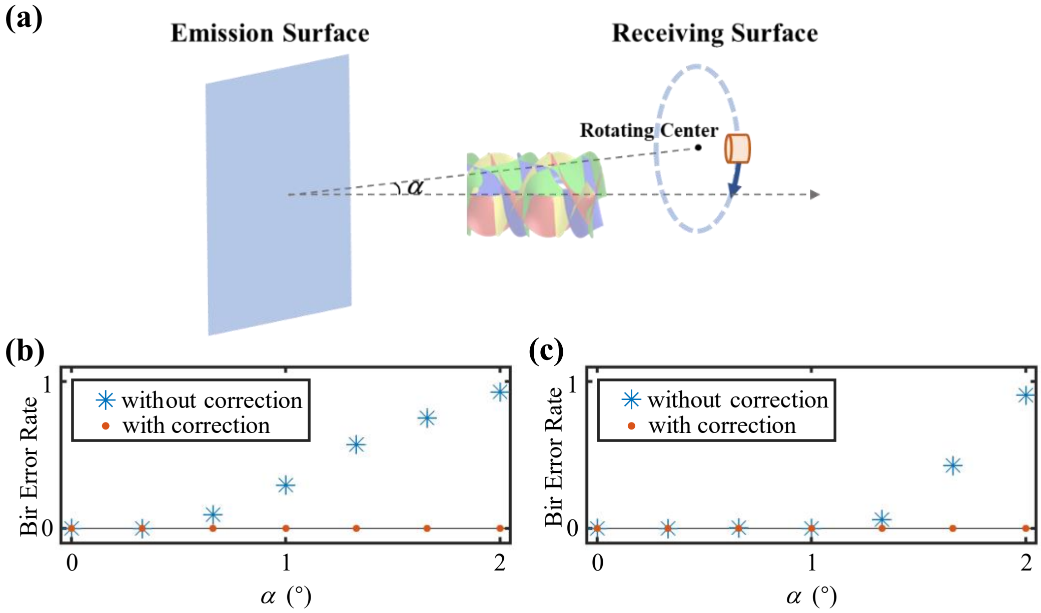

Figure S3. Influence of alignment on transmission accuracy of the OAM multiplexing communication under (b) ASK and (c) PSK modulations. The misalignment degree is determined by the deviation angle α.
